# Effects of repeated sleep deprivation on brain pericytes in mice

**DOI:** 10.1038/s41598-023-40138-0

**Published:** 2023-08-07

**Authors:** Yan Wu, Pengfei Li, Narayan Bhat, Hongkuan Fan, Meng Liu

**Affiliations:** 1 Department of Psychiatry and Behavioral Sciences, Medical University of South Carolina, Charleston, SC 29425 USA; 2 Pathology and Laboratory Medicine, Medical University of South Carolina, Charleston, SC 29425 USA; 3 Neuroscience, Medical University of South Carolina, Charleston, SC 29425 USA

**Keywords:** Neuroscience, Blood-brain barrier, Circadian rhythms and sleep, Neuro-vascular interactions

## Abstract

The damaging effects of sleep deprivation (SD) on brain parenchyma have been extensively studied. However, the specific influence of SD on brain pericytes, a primary component of the blood–brain barrier (BBB) and the neurovascular unit (NVU), is still unclear. The present study examined how acute or repeated SD impairs brain pericytes by measuring the cerebrospinal fluid (CSF) levels of soluble platelet-derived growth factor receptor beta (sPDGFRβ) and quantifying pericyte density in the cortex, hippocampus, and subcortical area of the PDGFRβ-P2A-CreER^T2^/tdTomato mice, which predominantly express the reporter tdTomato in vascular pericytes. Our results showed that a one-time 4 h SD did not significantly change the CSF sPDGFRβ level. In contrast, repeated SD (4 h/day for 10 consecutive days) significantly elevated the CSF sPDGFRβ level, implying explicit pericyte damages due to repeated SD. Furthermore, repeated SD significantly decreased the pericyte densities in the cortex and hippocampus, though the pericyte apoptosis status remained unchanged as measured with Annexin V-affinity assay and active Caspase-3 staining. These results suggest that repeated SD causes brain pericyte damage and loss via non-apoptosis pathways. These changes to pericytes may contribute to SD-induced BBB and NVU dysfunctions. The reversibility of this process implies that sleep improvement may have a protective effect on brain pericytes.

## Introduction

Sleep regulates the blood–brain barrier (BBB) permeability and promotes the clearance of metabolites^1–3^. For instance, amyloid-beta (Aβ) clearance is more effective during sleep than during waking^4^. Conversely, sleep loss or prolonged waking impairs the function of BBB and becomes a risk factor for Aβ accumulation in Alzheimer’s disease^5–7^. Recent findings also suggest that sleep, especially slow-wave sleep (SWS) or non-rapid eye movement (NREM) sleep, orchestrates cerebral blood flow (CBF) and cerebrospinal fluid (CSF) oscillations for metabolite clearance^8,9^. However, the exact cellular mechanism mediating sleep’s effects on BBB and other neurovascular events is still unclear. In other words, the bridge connecting the characteristic neuronal and vascular oscillations during SWS remains unknown.

**Figure 1:**
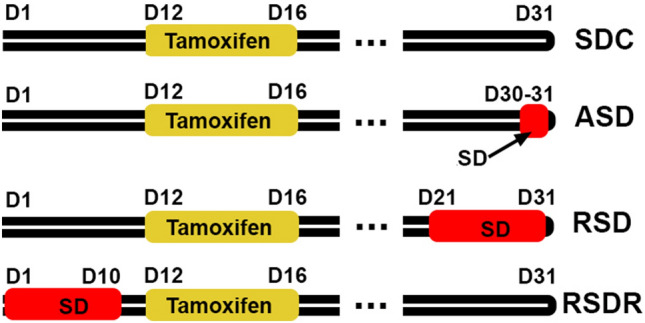
Experiment flowchart: The ASD group received one time SD on Day 30; the RSD group received 10 days SD between Day 21–30; the RSDR group received 10 days SD between Day 1–10; No SD was imposed on the control group (SDC). All mice received tamoxifen injections between Day 12–16 and were sacrificed two weeks after the tamoxifen injection (Day 31). Thus, the duration of tdTomato expression was the same across all groups.

Brain mural cells include pericytes and vascular smooth muscle cells (vSMCs). Pericytes are a crucial component of the neurovascular unit (NVU) and BBB that play a vital role in regulating CBF, maintaining BBB integrity, releasing neurotrophic factors, and other yet-to-be-understood functions^10–13^. The constriction and dilation of pericytes control the CBF fluctuation, which forms the basis of BOLD (blood-oxygen-level-dependent) functional imaging tools such as functional MRI (fMRI) and PET (positron emission tomography), to predict neuronal activity changes^14–16^. Since CBF and BBB functions are regulated by sleep^8–10,17,18^, it is intriguing to ask whether pericytes are one of the cellular targets of sleep in meditating its functions such as metabolite clearance and maintaining BBB integrity and whether sleep disruption/loss impairs brain functions through damaging pericytes. So far, minimal studies have associated sleep or circadian rhythm with pericytes. One study demonstrated that knocking out clock gene bmal1 (brain and muscle Arnt-like protein-1) causes severe loss of pericytes and profound BBB permeability changes^19^, implying the importance of circadian rhythm in pericyte health. A recent study showed that expressing the bmal1 gene in pericytes promotes vessel maturation in a 3D tissue scaffold model^20^. The other study showed that rapid eye movement (REM) sleep loss in rats induces pericyte detachment from the capillary walls^21^. The present study thoroughly assesses the pericyte damage caused by acute (ASD, one-time, 4 h) and repeated sleep deprivation (RSD, 4 h/day for 10 consecutive days) with a model that deprives both REM and NREM sleep by CSF platelet-derived growth factor receptor beta (PDGFRβ) measurement and a flow cytometry-based pericyte quantification method. A recovery group (RSDR, 3 weeks recovery after RSD) was included to examine whether the SD-induced pericyte changes were reversible (Fig. 1, flowchart).

## Results

### Distribution of tdTomato fluorescence in the brain of the Pdgfrβ-P2A-CreERT^2^/tdTomato mice

**Figure 2:**
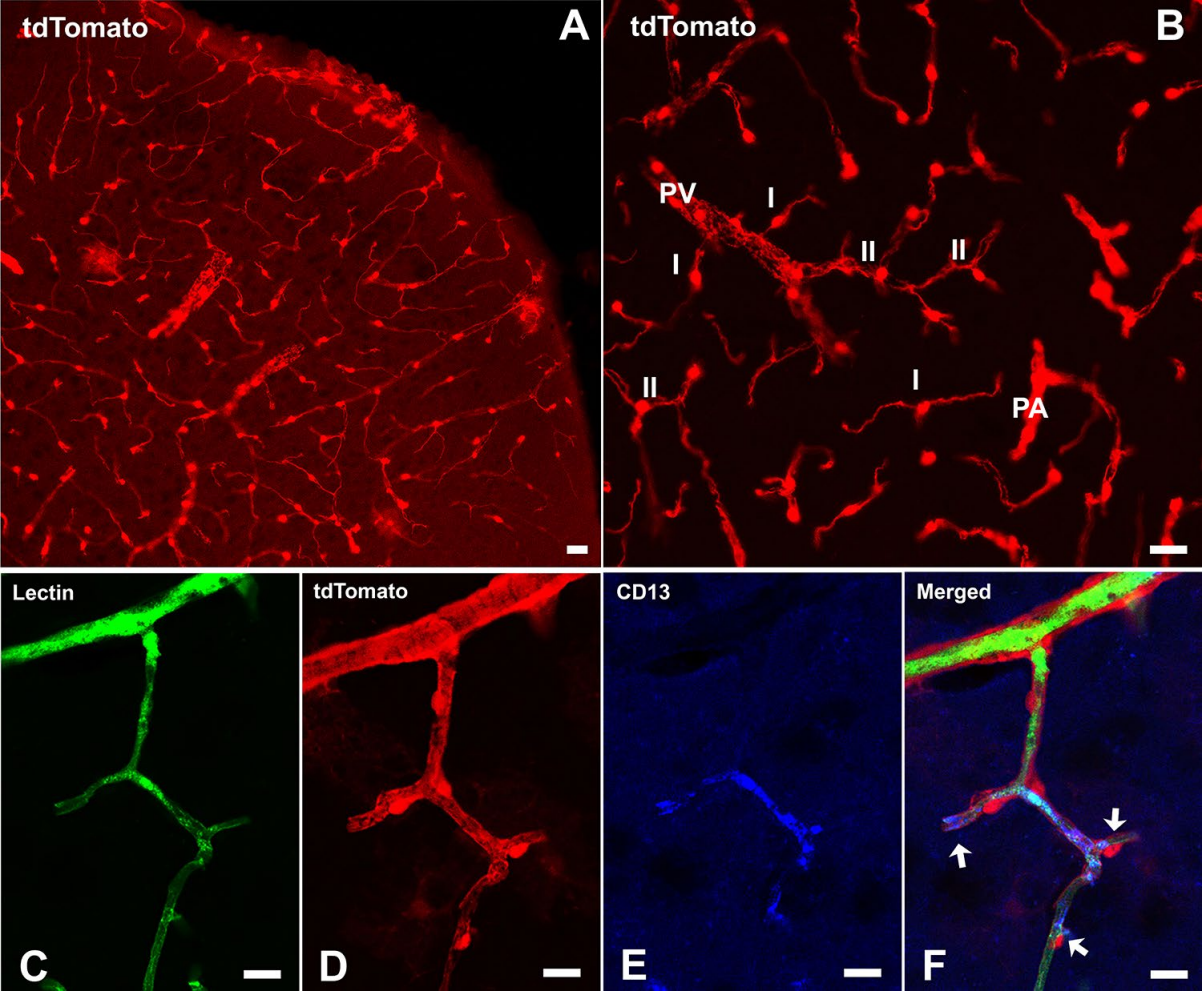
(**A**–**B**) Distributions of tdTomato^+^ pericytes in the cortex of a mouse in the control group. I: Single-strand pericytes; II: Mesh pericytes. PA: Penetrating arteriole; PV: Penetrating vein. (**C**-**F**): Blood vessels labeled with lectin (green), tdTomato (red), and CD13 (blue). Arrows in panel (**F**) indicate CD13^+^/tdTomato^+^ capillary pericytes. Scale bar = 25 µm.

Pdgfrβ-P2A-CreERT^2^/tdTomato mice express reporter tdTomato in vascular pericytes after tamoxifen induction. Consistent with other studies using similar PDGFRβ promoter-based mouse models, the tdTomato fluorescent signal induced by tamoxifen injection showed predominant perivascular localizations that include arterioles, capillaries, and venules in the mouse brain^22–24^. However, the protruding tdTomato^+^ ovoid somata characteristic of pericytes were predominantly distributed in the capillary, pre-capillary arteriole, and post-capillary venule wall. Capillary pericytes outlined by tdTomato fluorescence either extend a thin process around the vessel lumen (thin-strand pericytes) or have mesh-like processes (mesh pericytes). TdTomato^+^ somata were rarely observed in the brain parenchyma, penetrating arteriole, and ascending venule (Fig. 2A,B).

**Figure 3:**
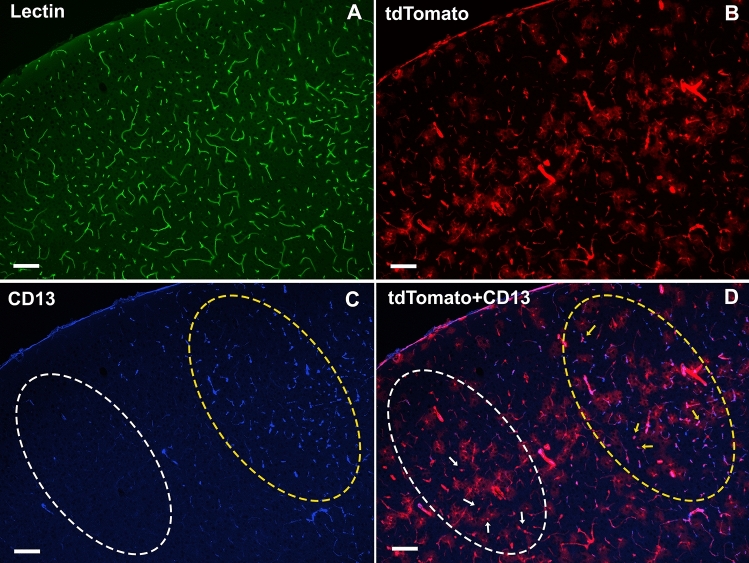
The discrepancy of CD13 immunostaining and tdTomato expression in terms of pericyte labeling. Panel (**C**) demonstrates dense CD13 immunoreactivities in the yellow-circled area in the motor cortex of a control mouse. However, CD13 immunoreactivities were little or undetectable in the adjacent white-circled area, though both areas had a similar amount of lectin (**A**) and tdTomato (**B**) distributions. Yellow arrows show the CD13^+^/tdTomato^+^ cells. White arrows indicate the CD13^-^/tdTomato^+^ cells. Scale bar = 50 µm.

To verify the pericyte-specificity of tdTomato expression, we examined the co-labeling of the tdTomato with immunostaining of CD13 (Aminopeptidase N), a primary surface marker for brain pericytes^25,26^. In brain sections, the distribution of CD13 immunoreactivities varied. Evident CD13 immunoreactivities were present in most brain areas, where about 98% of the CD13^+^ cells also expressed tdTomato (CD13^+^/tdTomato^+^) (Fig. 2C,F). However, we found that CD13 immunoreactivities were absent or very weak in some cortical and subcortical areas where numerous tdTomato^+^ cells and lectin-labeled blood vessels were present (Fig. 3). Since capillaries and pericytes were unlikely absent in such extensive brain areas, we believe that the negative CD13 labeling in these brain areas was probably due to the limitation of the immunostaining technique, which may not detect low-level CD13 expression. The same distribution pattern also applied to the PDGFRβ immunostaining (see Supplementary Fig. S1). Therefore, in the following flow cytometry study, we used the genetic reporter tdTomato rather than CD13 for pericyte quantification to avoid underestimation of pericyte densities.

### Effects of SD on CSF sPDGFRβ level

**Figure 4:**
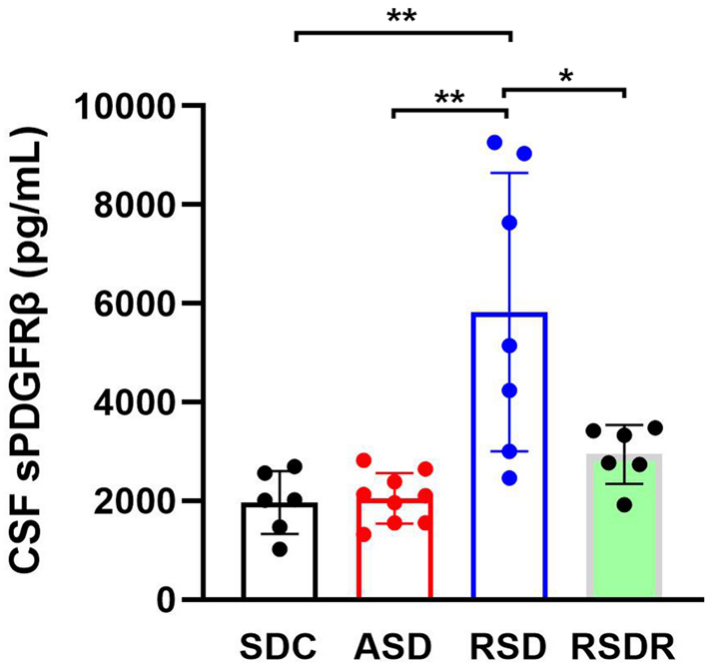
*ELISA* assay results showed that the CSF sPDGFRβ level in the RSD group was significantly higher sPDGFRβ compared to other groups (**: *p* < 0.001; *: *p* < 0.05).

Injured brain pericytes shed sPDGFRβ into CSF. Therefore, an elevated CSF sPDGFRβ level has become a sensitive marker for pericyte injury. The CSF sPDGFRβ levels in the control group (SDC), ASD, RSD, and RSDR groups were measured with ELISA assay. The average CSF sPDGFRβ level in the SDC mice was 1964 ± 638.0 pg/mL, nearly half of the young human CSF sPDGFRβ level^27^. ANOVA analysis showed significant differences in sPDGFRβ level among four groups (*F*_3,24_ = 10.39, *p* = 0.0001). ASD did not significantly change the CSF sPDGFRβ level (2052 ± 513.9 pg/mL, t = 0.11 dF = 24, *p* > 0.99 compared to the SDC group). However, RSD dramatically increased CSF sPDGFRβ level, nearly threefold higher than the SDC group (5827 ± 2821.0 pg/mL. t = 4.64 and 5.01 compared to SDC and ASD groups, respectively, dF = 24, *p* < 0.001). A 3 weeks recovery decreased the CSF sPDGFRβ level down to the baseline level (2945 ± 595.3 pg/mL, t = 1.14, dF = 24, *p* > 0.99 compared to the SDC group) (Fig. 4). Unchanged CSF sPDGFRβ level in ASD indicated that a one-time 4-h SD did not cause noticeable damage to pericytes. Therefore, we did not include the ASD group in the following flow cytometry experiments to minimize the number of animals used.

### Expression of cleaved caspase-3 in pericytes

**Figure 5:**
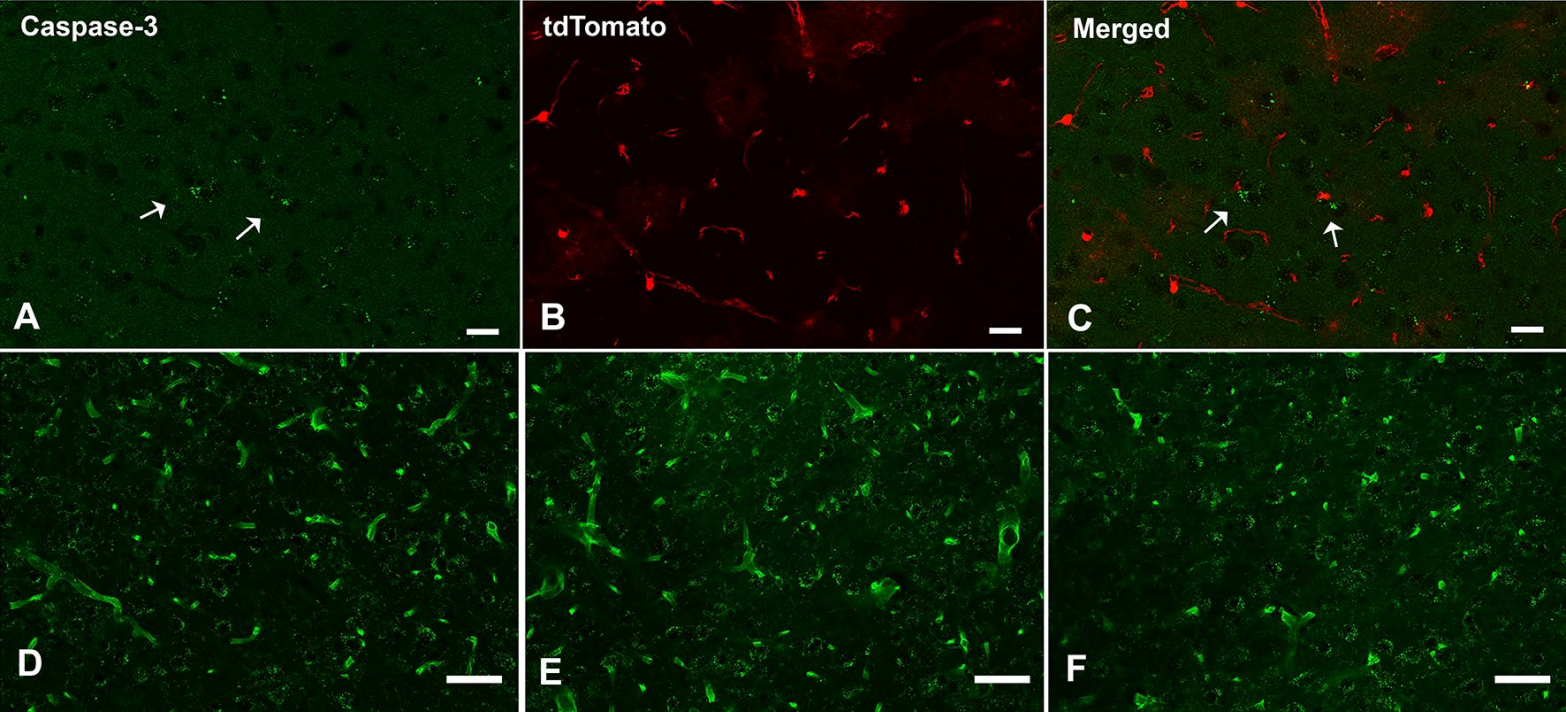
(**A**–**C**) Cleaved Caspase-3 Immunostaining results in the cortex of an RSD mouse. Positive immunoreactivities (arrows) were observed in the brain parenchyma but did not co-localize with tdTomato fluorescence. (**D**–**F**) Lectin staining in the cortex of SDC (**D**), RSD (**E**), and RSDR (**F**) mice, respectively. No significant vascular density change was observed. Scale bar = 50 µm.

Cleaved Caspase-3 is a valuable marker for apoptosis and usually cannot be detected in healthy cells. Increased cleaved Caspase-3 immunoreactivities in the cytoplasm of brain parenchyma cells were observed in the RSD mice, but the cleaved Caspase-3^+^/tdTomato^+^ cells were rarely observed in all groups, implicating that there was no pronounced pericyte apoptosis induced by repeated SD (Fig. 5A–C, Supplementary Fig. S5).

### Effects of repeated SD on brain vascular density:

Lectin (Lycopersicon esculentum) is commonly used to visualize blood vessels due to its ability to bind to the basal membrane of endothelial cells (ECs). The percentage of the area covered by Lectin staining is a reliable index of vascular density and has been used to quantify blood vessels in many studies. We did not find significant group differences in vascular area densities in the cortex and hippocampus among all three groups, indicating that our SD procedure did not significantly change the overall structure of brain blood vessels or cause severe EC damage/loss (Fig. 5, D-F; Supplementary Fig. S2).

### Effects of repeated SD on tdTomato^+^ pericyte density: results of flow cytometry assay

TdTomato fluorescence in the brain was potent and all over the vasculature system, making some tdTomato^+^ somata unrecognizable, which will introduce bias if we count pericytes on brain sections with the traditional morphometry method. Therefore, we applied the flow cytometry method to quantify pericytes in three brain areas (cortex, hippocampus, and subcortical areas). In the SDC group, about 5.33 ± 1.19% of hippocampus cells, 10.53 ± 0.84% of cortical cells, and 9.68 ± 3.64% of subcortical cells were tdTomato^+^ cells. The pericyte density range we obtained is much higher than that of Crouch’s 2018 study (~ 2%) but comparable with Spitzer’s 2013 study (10–20%)^26,28^. Factors such as tissue isolation procedures, digestive protease concentration, and antibody selection can impact flow cytometry results. To enable meaningful comparisons, we ensured that all experimental conditions were consistent across all groups.

**Figure 6:**
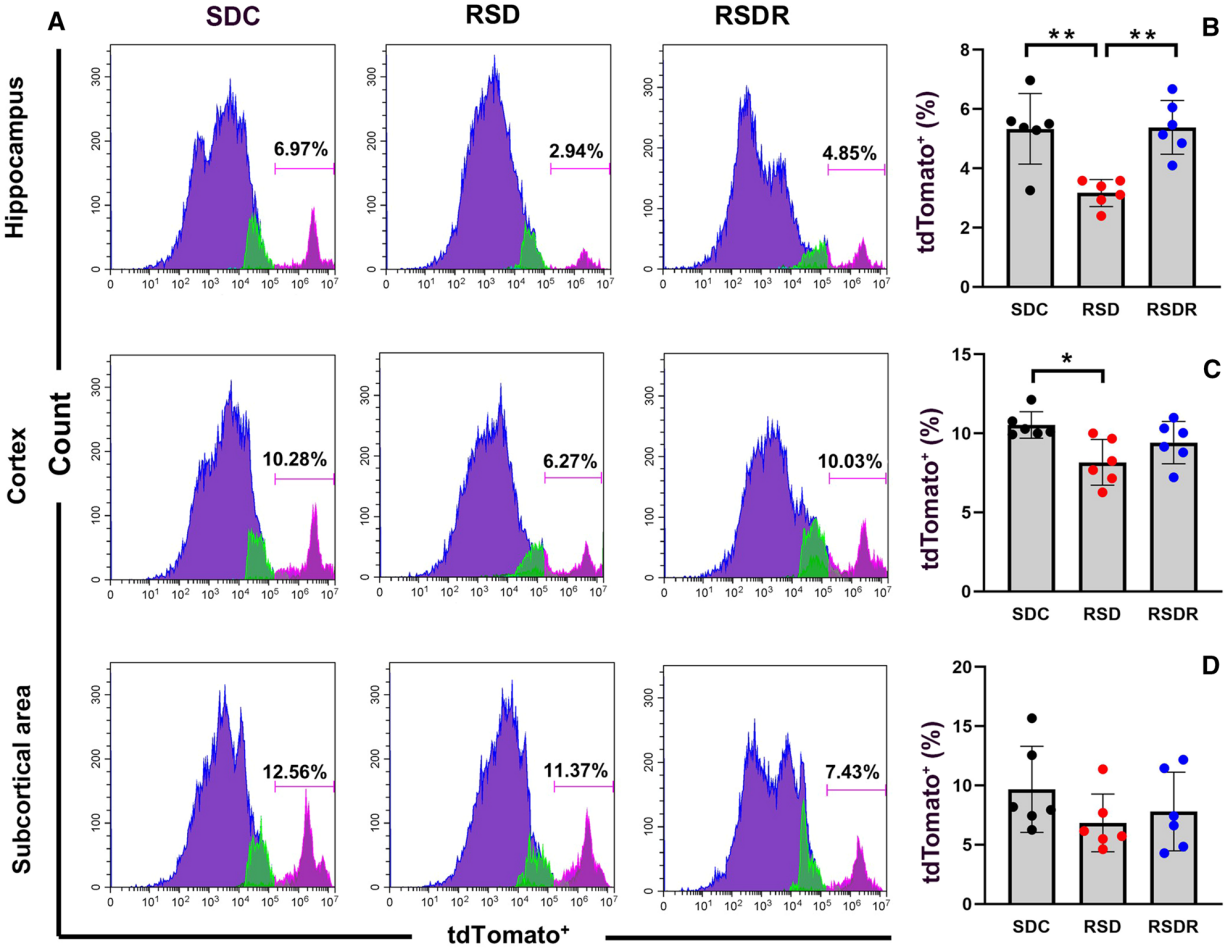
(**A**) Representative photos of pericyte (tdTomato^+^) densities measured with flow cytometry in three regions. (**B**–**D**) Comparisons of pericyte density among three groups in different brain areas. RSD significantly reduced the pericyte densities in the hippocampus (**B**) and cortex (**C**) (*: *p* < 0.05; **: *p* < 0.01). However, after a 3-week recovery, pericyte densities in the hippocampus and cortex were restored, with no significant difference from the SDC control group.

**Figure 7:**
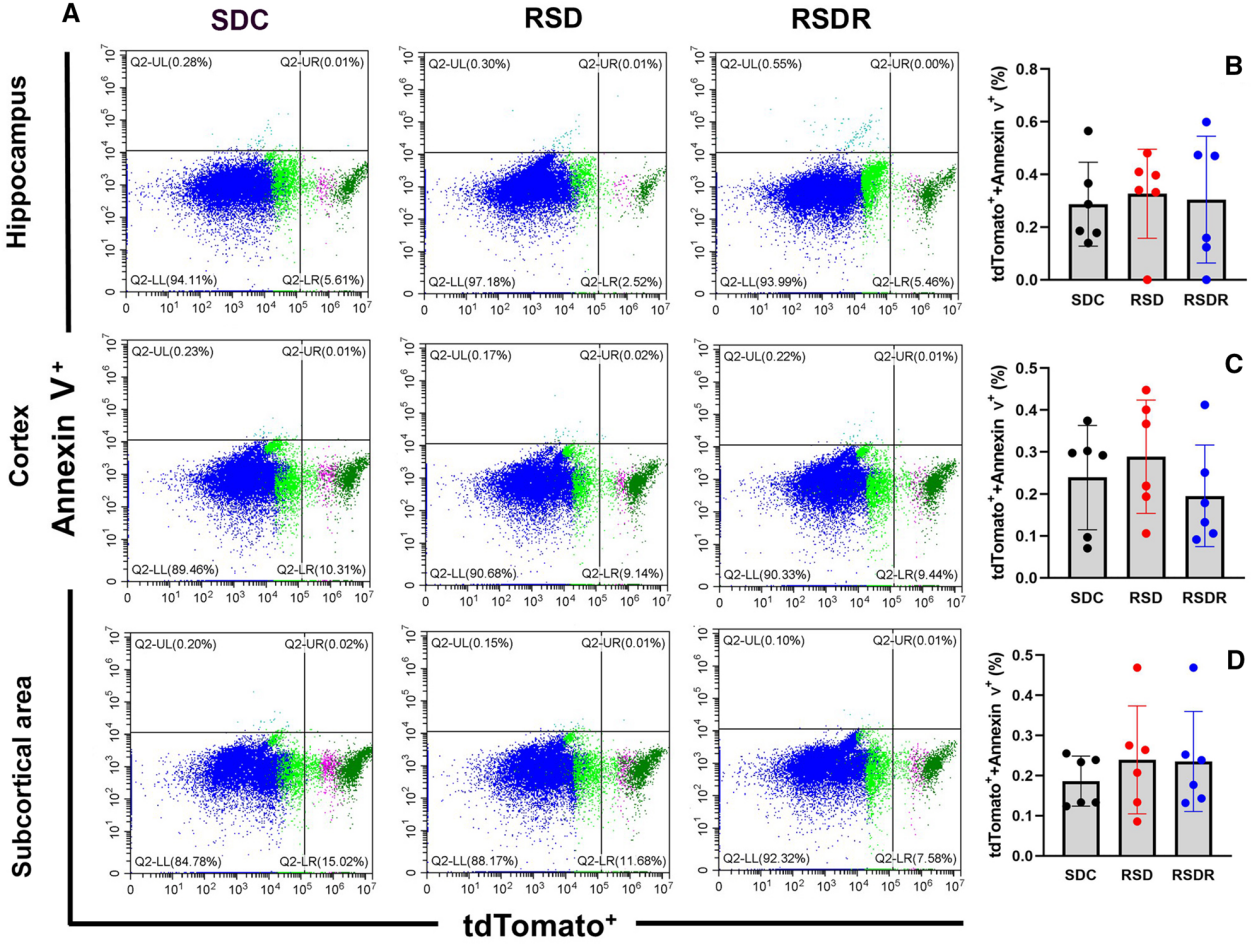
(**A**) Representative photos of ratios of tdTomato^+^/Annexin V^+^ cell to tdTomato^+^ cell measured with flow cytometry in three brain areas. (**B**–**D**) Comparison of the densities of apoptotic pericytes(tdTomato^+^/Annexin V^+^). No significant difference was found among all three groups.

Ten days repeated SD significantly decreased tdTomato^+^ cell densities in the hippocampus to 3.17 ± 0.46% (t = 4.15 dF = 15, *p* = 0.0026) and in the cortex to 8.17 ± 1.45% (t = 3.30, dF = 15, *p* = 0.015). The tdTomato^+^ cell densities in the subcortical area did not differ significantly among the three groups. After a 3-week-long recovery from repeated SD, densities of tdTomato^+^ cells in the cortex and hippocampus were restored to the baseline level (Fig. 6). The densities of apoptotic pericytes (tdTomato^+^/Annexin V^+^) were very low in all three brain regions, and the differences in apoptotic pericyte density (ratio of tdTomato^+^/Annexin V^+^ cells to tdTomato^+^ cells) among each group were insignificant (Fig. 7).

## Discussion

Brain vascular mural cells include smooth muscle cells (vSMC) and pericytes. Pericytes are highly abundant in the microvasculature in the central nervous system (CNS). Damage or loss of brain pericytes has been reported in various CNS disorders, including ischemic stroke, Alzheimer’s disease (AD), and diabetic retinopathy^29–33^. Our study demonstrated that repeated sleep deprivation (SD) impaired pericytes at both molecular and cellular levels.

SD is a traditional approach to studying the function of sleep and the effects of insomnia or prolonged waking in animals. However, commonly used methods such as gentle handling, wheel-running, disk-over-water, or multiple-platform often result in partial SD (such as REM SD only) or frequent awakening/arousal, excessive locomotion activities, and unavoidable systematic stress that confound the real effects of sleep loss^34–37^. To address these drawbacks, the rotating bar-based automated SD system has emerged. The remote control of the rotating bar minimizes the experimenter-induced stress, while the adjustable rotating speed prevents animals from drifting into sleep. Furthermore, our rotating bar system is unique in that the mice do not need to walk over the bar actively; instead, the height of the bar puts gentle nudges on the head or shoulder to awaken animals without notably increasing their locomotion (see Supplementary Fig. S4). As a result, the changes to pericyte found in our study can be solely attributed to SD rather than locomotion. A 24 h EEG/EMG recording was performed to validate our SD system (see Supplementary Figs. S3 and S4).

In mice, PDGFRβ is predominantly expressed in pericytes^38^, while in humans, both pericytes and vSMCs express PDGFRβ, with the relative protein abundance of PDGFRβ four times higher in pericytes than in vSMCs^39^. Importantly, PDGFRβ in pericytes, but not in vSMCs, is cleaved and shed into CSF during hypoxia and other injuries. Thus, elevated CSF PDGFRβ has become a sensitive pericyte injury biomarker associated with BBB breakdown and early cognitive dysfunction in humans^27,31,39–41^. As far as we know, this is the first time that CSF sPDGFRβ was measured in mice.

One-time 4-h ASD failed to increase the level of sPDGFRβ. However, RSD caused a threefold increase in the sPDGFRβ level in CSF, suggesting RSD-induced severe pathological changes in pericytes, including shedding degraded membrane receptor PDGFRβ into CSF. Since platelet-derived growth factor (PDGF), the endogenous ligand of PDGFRβ, is secreted by the ECs, the rapid and massive loss of the surface receptor PDGFRβ may disrupt the normal crosstalk between pericytes and the ECs, which is crucial for pericyte proliferation, migration, and hence may eventually result in pericyte death^42–44^. Nevertheless, mice given a 3 weeks recovery period after the same 10 days repeated SD procedure managed to maintain the CSF sPDGFRβ level at the baseline level, indicating that pericytes have stopped PDGFRβ shedding after SD was abolished.

Sleep is essential for various physiological functions and homeostasis. The modern understanding is that a complex neural circuitry in the brain regulates the sleep/wake states, under the control of two internal biological mechanisms—circadian rhythm and homeostasis^45^. However, growing evidence indicates that many non-neuron elements, such as the glial cells, microvascular system, BBB, and the immune system, also impact neural circuits or local neurons and significantly contribute to sleep regulation and sleep homeostasis^3,46–48^.

Therefore, SD could damage not only neural substrates but also these non-neuron elements, which may include the brain pericytes, as they are a vital component of the BBB and NVU. However, direct evidence of the interactions between sleep and pericytes is scarce. Indirect evidence suggests that BBB permeability changes caused by circadian rhythm and sleep loss are mediated by pericytes^1,3,10,17,18^. So far, only one published study has examined the direct effect of SD on pericytes, in which they found that SD disrupted the interaction between pericytes and ECs and decreased microvascular PDGFRβ levels in rat brain tissue^21^. However, the multiple-platform model used in this study produced a partial SD that reduces most REM sleep but only a portion of NREM sleep^49^.

Because the potential involvement of pericytes in generating CBF and CSF oscillations happens mostly during NREM sleep (or slow-wave sleep)^8,50^, we postulate that an SD procedure that produces the loss of both NREM and REM sleep may have a greater impact on pericytes. Indeed, we demonstrated that our repeated SD model resulted in significant pericyte loss in the cerebral cortex and hippocampus, accompanied by increased CSF sPDGFRβ levels. These changes will inevitably contribute to BBB or NVU damage observed in SD.

The mechanisms mediating these pericyte losses or abnormalities are still unclear. Previous studies have shown that brain or retinal pericytes undergo apoptosis under certain conditions, such as high glucose and Alzheimer’s disease^29,51,52^. However, SD-induced pericyte loss may have different mechanisms since our study failed to detect any significant signals of pericyte apoptosis. Consistent with our results, Medina-Flores’s study also found no change in the expression level of activated Caspase-3 in isolated brain microvessels of sleep-deprived rats. Necrosis (non-apoptotic cell death) is the other primary mechanism of cell death, which could be visualized by Annexin V^−^/PI^+^ staining in the flow cytometry study. However, our data were unsuitable for calculating SD-induced necrosis because some of these Annexin V^−^/PI^+^ cells may die from the tissue preparation procedures.

In addition to cell death, SD-induced pericyte loss may be attributed to the transition of pericytes to other types of cells. Studies have shown that pericytes have the potential to differentiate into other tissue-specific types of cells under certain circumstances in vitro and in vivo^53–56^. For instance, brain pericytes can acquire a microglia phenotype by expressing microglia markers such as IBA1 after an ischemic stroke^57^. Therefore, more studies are needed to explain the exact mechanism of SD-induced pericyte loss.

Vascular ECs are another significant component of BBB. Although multiple studies have shown that SD can affect endothelial functions and contribute to cardiovascular dysfunctions such as hypertension^58,59^, SD-induced substantial EC damage or loss has not been reported. In fact, massive EC death or loss only occurs under severe brain injuries such as ischemic stroke or neurodegenerative ataxia when the microvascular breakdown occurs^60,61^. We did not detect any significant changes in the vascular density in the cortex and hippocampus by comparing the percentages of Lectin-stained areas among the three groups. This finding suggests that our repeated SD procedure did not cause notable changes in ECs and the overall blood vessel structure. It also implies that pericytes are more sensitive to SD than ECs, and the pericyte loss induced by repeated SD was not a consequence of a massive loss of microvessels.

The other important finding of the present study is that the pericyte damage and loss caused by 10 days repeated SD could be reversed if the normal sleep/wake cycle was reinstated. The pericyte’s strong capacities for proliferation and repair could explain this reversal^42^. These capacities are also crucial for microvasculature repair and maintaining the BBB and NVU integrity under other physiological and pathological conditions.

Instead of using a total or extended SD model, the present study used a moderate SD model (4 h/day) because, clinically, mild to moderate SD is more common than total SD in everyday life^62^. Thus, we do not know whether a more severe SD (> 4 h/day and > 10 days) or a total SD could cause irreversible pericyte damage or loss. Another limitation is that we did not measure the SD-induced BBB permeability changes. Therefore, we can not examine the potential correlations between pericyte loss or elevated sPDGFRβ level and BBB damage. Future studies should test an extended SD model and examine simultaneous changes in markers of other NVU or BBB components. In addition, an animal model that can be used to precisely target CNS capillary pericytes is needed to study the complex cellular interactions within NVU and BBB during normal or pathological sleep/wake regulation.

## Conclusion

We identified cell damage and loss induced by repeated SD on brain pericytes, a critical component of brain vascular mural cells. These pericyte changes are likely to contribute to BBB breakdown and dysfunction in brain microcirculation during chronic SD. Conversely, our findings suggest that sleep improvement could protect pericytes and guard against pericyte-related pathological conditions, such as Alzheimer’s disease, in the brain.

## Materials and methods

### Animals

We crossed the Pdgfrβ-P2A-CreER^T2^ (Jax # 030201)^24^ and the Ai14 tdTomato reporter mice (Jax #007914) to obtain the Pdgfrβ-P2A-CreER^T2^/tdTomato mice, in which reporter tdTomato will be expressed primarily in pericytes after induction with tamoxifen injection (75 mg/kg body weight, i.p. once every 24 h for a total of 5 consecutive days). Animal breeding and manipulations followed the policies established in the National Institutes of Health Guide for the Care and Use of Laboratory Animals and the Institutional Animal Care and Use Committee (Protocol # IACUC-01399). All mice were housed in a 12 h/12 h light/dark environment with lights on at zeitgeber time 0 (ZT00) and fed ad libitum standard rodent chow and water. Ambient temperature and humidity were maintained between 22 and 24 °C and 40–60%, respectively. Two cohorts of 6–8 months-old PDGFRβ-P2A-CreER^T2^/tdTomato mice (including both sexes) were randomly assigned to the control group (SDC), acute SD group (ASD), 10 days repeated SD group (RSD), and recovery group (RSDR, RSD+ 3 weeks recovery), with 5–9 mice in each group. The time frame of tamoxifen induction, SD procedures, and tissue harvest is listed in Fig. 1. One cohort of mice was used for CSF collection and histology; the other cohort was used for flow cytometry.

### SD procedures

Mice were sleep-deprived using an automated sleep deprivation system with a motorized rotating bar on the cage floor. The effectiveness of similar devices for producing SD has been previously validated^63,64^. Briefly, mice were group-housed in cages containing a rotating bar during the experiment. All mice were acclimated to these new cages by turning on the rotating bar (6–10 rpm) for 5 min per hour during the dark (active) phase. Then a 4 h ASD (ZT00-04) or a 10 days RSD (4 h/day, ZT00-04) was produced by activating the rotating bar continuously in the designated time frames. The rotating bar gently nudges and prevents mice from falling asleep without significantly increasing their locomotor activities. The rotating bar remained still during the light (inactive) phase for the control mice. Mice’s behaviors during SD were videotaped to confirm that SD procedures were undisrupted and mice were kept awake throughout the whole SD procedure.

### CSF collection and sPDGFRβ ELISA assay

Mouse CSF was collected as described in Lim’s protocol^65^. Briefly, under anesthesia (1% isoflurane inhalation), the cisterna magna was exposed by removing nearby muscles. Then, a clean glass capillary was punctured into the cisterna magna, and CSF was automatically drawn into the capillary tube. We obtained 8–15 µl CSF per mouse. To avoid the possible sPDGFRβ concentration fluctuations due to circadian time, we always collect CSF samples at ZT05-06. Then, the standard sandwich ELISA assay was performed according to the manufacturer's protocol (Abcam, Cambridge, MA). The plate was read immediately on the BioTek Synergy 4 reader. Soluble PDGFRβ (sPDGFRβ) concentrations were calculated using the samples’ readings and the linear standard curve equation. The results were multiplied by the dilution factor to arrive at the final concentration in the original CSF samples.

### Pericyte density quantification with flow cytometry

Flow cytometry was performed to quantify pericytes in mice brain tissue based on published protocols^26,28^. Briefly, mice were perfused with 10 mL PBS, and the bilateral cerebral cortex, hippocampus, and subcortical area (between the inferior colliculus and anterior corpus callosum) were harvested. Brain tissue was flushed with 2% fetal bovine serum/phosphate-buffered saline (FBS/PBS), cut into 1 mm–long pieces, and incubated in complete medium containing 3 mg/mL collagenase/dispase (Sigma, St Louis, MO) for 45 min at 37 °C with continuous shaking. Brain tissue was then triturated (pipette up and down) ∼100X with a P1000 pipette, and the supernatant was strained and centrifuged at 300*g* for 5 min. Pellets were resuspended in 22% Percoll (Sigma, St Louis, MO) and centrifuged at 2600*g* for 10 min. Blood cells were removed by red blood cell lysis buffer (Sigma, St Louis, MO) and a 40 μm cell strainer. Pellets were resuspended in PBS with 2% FBS/PBS, and cell density was measured. Multi-color flow cytometry was performed according to standard techniques with a CytoFLEX LX (Beckman Coulter) using filters for tdTomato (pericytes), Annexin V-Pacific Blue (Thermofisher, Waltham MA, for apoptosis), and Propidium iodide (PI, Thermofisher, Waltham MA, for dead cells).

### Immunofluorescence staining and image analysis

Immediately after CSF collection, mice were transcardially perfused with PBS solution containing 10% formalin, and the brains were cross-sectioned at 40 µm thickness on a compresstome (Precisionary Instruments, Greenville, NC). Brain sections were divided into four sets. One set of sections was stained with Lectin-DyLight 488 (1:200 dilution, Thermofisher, Waltham MA) and rabbit anti-CD13 monoclonal antibody (ab32570, 1:4000 dilution, Abcam, Cambridge MA) to verify tdTomato-expressing cell identities and locations. Other sections were stained with rabbit anti-cleaved Caspase-3 monoclonal antibody (1:1000 dilution, Cell Signaling, Danvers, MA) to detect apoptotic cells. Alexa Fluor-647 donkey anti-rabbit IgG was used as the secondary antibody. For quantitative analysis of the vascular density, 8 Lectin-stained coronal Sections (2 at AP + 0.6; 2 at AP-0.9; 2 at AP-1.6; 2 at AP-2.3 level) were scanned, and high-definition images were taken using Zeiss LSM 880 confocal microscope. Images were then transferred to NIH ImageJ software. Based on fluorescent intensities ranging from 0 to 255, blood vessels were distinguished from background signals by setting a threshold at 30, which was constant in all three groups. The cortical areas covering the primary and secondary motor cortices, the primary and secondary somatosensory cortices, and the whole hippocampal structure were examined. The vascular area density was determined by the area ratio of the Lectin-stained area to the entire area of interest^66,67^.

### Statistical analysis

Data were analyzed using GraphPad Prism 9.2 (GraphPad Software, Boston, MA). One-way ANOVA with Bonferroni post-hoc test was used to compare CSF sPDGFRβ level, pericyte density, and vascular area density among groups. Statistical significance was evaluated at the *p* < 0.05 (two-tailed) level^68^.

### Ethical approval

All animal care and procedures followed the policies established in the National Institutes of Health Guide for the Care and Use of Laboratory Animals and were reported in accordance with ARRIVE guidelines. All manipulations done to the mice were approved by the Medical University of South Carolina Institutional Animal Care and Use Committee (protocol # IACUC-2021-01399).

### Supplementary Information

Supplementary Figures.

## Data Availability

All data generated or analyzed during this study can be obtained from the corresponding author upon reasonable request.
