# Supplementary material for: Effects of repeated sleep deprivation on brain pericytes in mice
Source: Sci Rep. 2023 Aug 7;13:12760. doi: 10.1038/s41598-023-40138-0 (PMC10406921; doi:10.1038/s41598-023-40138-0)
Supplement: Supplementary file 1 — Supplementary Figures. [file 41598_2023_40138_MOESM1_ESM.pdf]

Supplementary Material

Effects of repeated sleep deprivation on brain pericytes in mice

Yan Wu[1], Pengfei Li[2], Narayan Bhat [3], Hongkuan Fan[2], Meng Liu[1*]

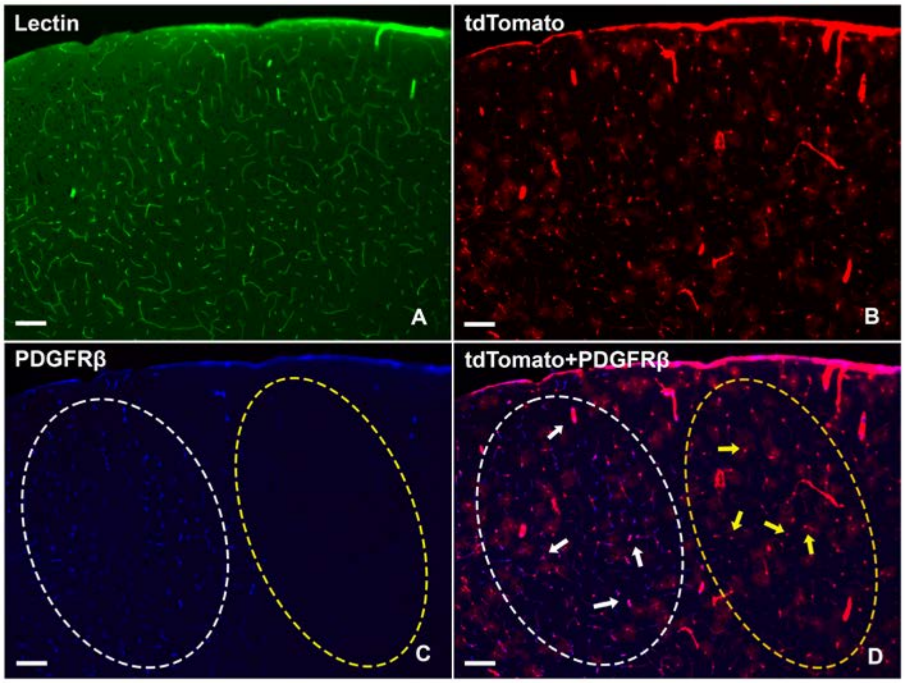

Figure S1. PDGFRβ immunostaining results in the cortex of a control mouse. PDGFRβ immunoreactivities, which were densely distributed in the white-circled area, were very weak or undetectable in adjacent yellow-circled area, though both areas had a similar amount of lectin (A) and tdTomato (B) distributions. White arrows show the PDGFRβ$^+$/tdTomato$^+$ cells. White arrows indicate the PDGFRβ$^-$/tdTomato$^+$ cells. Scale bar=50 µm. Mouse anti-PDGFRβ monoclonal antibody from Santa Cruz Biotech (1:2000) was used.

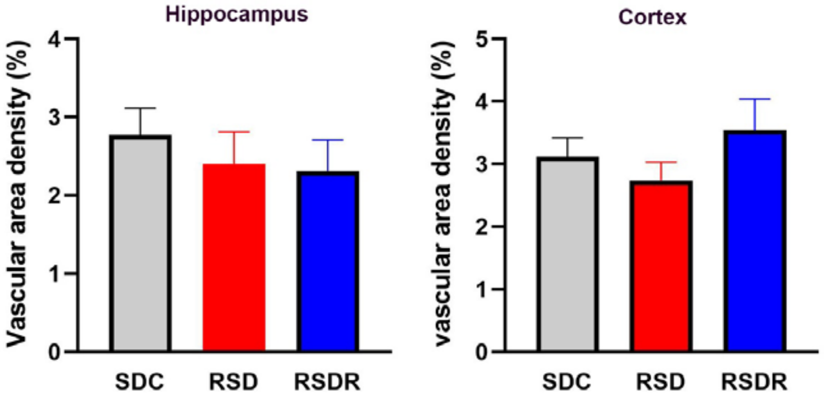

Figure S2. Vascular area density (Mean±SEM) in the hippocampus (Left) and cortex (Right). No significant difference was observed among all three groups.

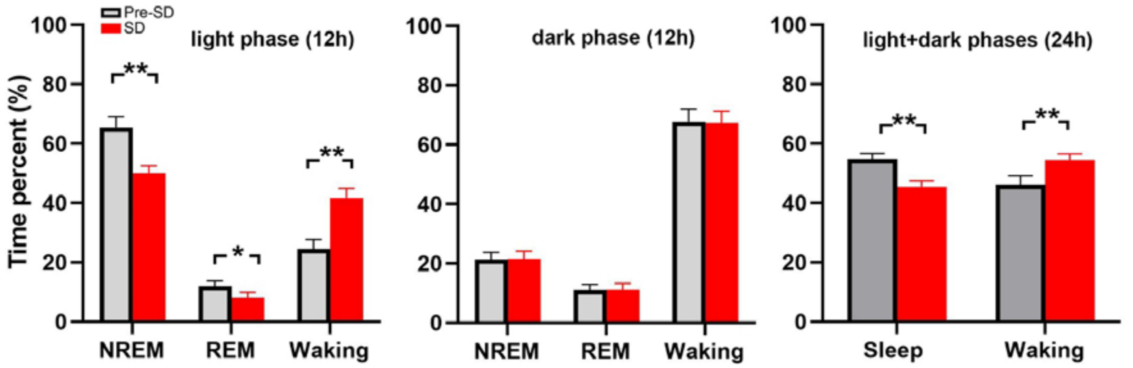

Figure S3. 5 Pdgfrβ-P2A-CreERT2/tdTomato (both sexes, age: 6~9 months) were installed with EEG/EMG electrodes. After recovery and acclimation to the SD cage, 24-h EEG/EMG recordings were performed for the day before SD (Pre-SD) and the SD day. EEG/EMG data were scored and analyzed with SleepSign software (Kissei Comtec, Japan) as described in (Liu et al., 2016). The total amounts of NREM sleep, REM sleep, and waking were compared between the Pre-SD and SD days. Our SD procedure caused significant sleep loss (*: $p<0.05$; **: $p<0.001$).

Liu, M., *et al.* (2016). Orexin gene transfer into the amygdala suppresses both spontaneous and emotion-induced cataplexy in orexin-knockout mice. *Eur J Neurosci*, *43*(5), 681-688,PMC4783302.

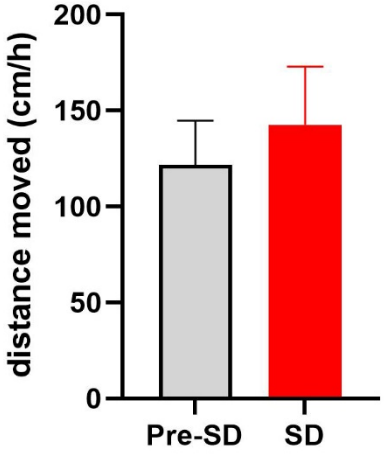

Figure S4. Recorded videos of the same 5 mice used for Fig. S3 were analyzed with Noldus EthoVision XT software. The distances traveled during ZT00-ZT04 were compared between pre-SD day and SD day. Mice traveled slightly more during SD procedure than control. However, the difference was statistically insignificant ($p>0.05$).

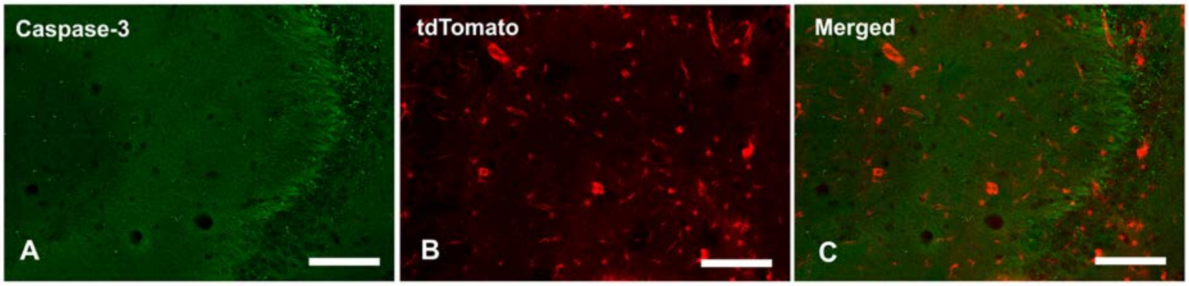

Figure S5. Caspase-3 Immunostaining results in the CA3 area of the hippocampus of an RSD mouse. Caspase-3 positive cells were rare and did not co-localize with pericytes (tdTomato+). Scale bar =100 µM
